# High-throughput screening identifies small molecules that enhance the pharmacological effects of oligonucleotides

**DOI:** 10.1093/nar/gkv060

**Published:** 2015-02-06

**Authors:** B. Yang, X. Ming, C. Cao, B. Laing, A. Yuan, M. A. Porter, E. A. Hull-Ryde, J. Maddry, M. Suto, W. P. Janzen, R. L. Juliano

**Affiliations:** 1 UNC Eshelman School of Pharmacy, University of North Carolina, Chapel Hill, NC 27599, USA; 2 Southern Research Institute, Birmingham, AL 35205, USA

## Abstract

The therapeutic use of antisense and siRNA oligonucleotides has been constrained by the limited ability of these membrane-impermeable molecules to reach their intracellular sites of action. We sought to address this problem using small organic molecules to enhance the effects of oligonucleotides by modulating their intracellular trafficking and release from endosomes. A high-throughput screen of multiple small molecule libraries yielded several hits that markedly potentiated the actions of splice switching oligonucleotides in cell culture. These compounds also enhanced the effects of antisense and siRNA oligonucleotides. The hit compounds preferentially caused release of fluorescent oligonucleotides from late endosomes rather than other intracellular compartments. Studies in a transgenic mouse model indicated that these compounds could enhance the *in vivo* effects of a splice-switching oligonucleotide without causing significant toxicity. These observations suggest that selected small molecule enhancers may eventually be of value in oligonucleotide-based therapeutics.

## INTRODUCTION

There is strong interest in the therapeutic potential of antisense oligonucleotides (ASO), siRNA and splice switching oligonucleotides (SSOs)(1–5). However, despite FDA approval of the first antisense drug (6) and the advent of multiple clinical trials (7–9), the development of oligonucleotides as therapeutic agents has progressed slowly. A major impediment has been the fact that delivery of these large, highly polar molecules to their sites of action in the cytosol or nucleus of cells in tissues is a very challenging problem (10–12).

There have been three broad approaches to the delivery of oligonucleotides. The most direct approach is to use well-designed molecules with chemical modifications to assure *in vivo* stability and high affinity binding to RNA targets (6,13,14). A second has been to incorporate oligonucleotides into various lipid-, polymer- or peptide-based nanocarriers (15–23). A third approach has been to covalently link oligonucleotides to ligands that interact with specific cell surface receptors thus promoting receptor-mediated endocytosis (24–34). However, difficult issues arise with all three approaches. Thus, most nanocarriers exhibit restricted delivery and are only effective in tissues where the vasculature is leaky, such as liver, spleen and some tumors (10,35). Additionally, the cationic lipids or polymers used in many nanocarriers have been associated with significant toxicities (36,37). Unmodified ‘free’ oligonucleotides, as well as ligand-oligonucleotide conjugates, are taken up by cells via endocytosis and accumulate in various endomembrane compartments where they are pharmacologically inert (38,39). Recent studies have shown that even in the case of lipid nanocarriers much of the oligonucleotide accumulated by cells remains entrapped in endosomes (40). Thus the biological effects of oligonucleotides may primarily be due to a small amount of material that escapes from endosomes and reaches key cytosolic or nuclear compartments.

Cells possess complex protein machinery that regulates endocytosis and subcellular trafficking (41–46). Recent work from our laboratory (24,26,47) and from others (31,48–50) has suggested that the route of cellular uptake and intracellular trafficking of an oligonucleotide can strongly influence its pharmacological action. This led us to hypothesize that we should be able to find small molecules that modulate intracellular trafficking so as to enhance oligonucleotide effects. However, despite the biological importance of these processes, there are only a few chemical tools available to manipulate endomembrane trafficking (51). One interesting example is a compound termed Retro-1 that influences the intracellular trafficking of bacterial and plant toxins (52). We found that Retro-1 could also enhance the effects of ASOs and SSOs (53). While the results with Retro-1 were encouraging, this compound is not ideal in that it is not very potent and is poorly water-soluble which makes *in vivo* studies difficult. Thus we turned to high-throughput screening of chemical libraries to discover novel small molecules that can enhance the pharmacological effects of oligonucleotides. Here we report the identification and characterization of a set of compounds capable of strongly enhancing oligonucleotide actions.

## MATERIALS AND METHODS

### Oligonucleotides, cell lines and other reagents

The 2′-O-Me phosphorothioate SSO SSO623 [5′-GTT ATT CTT TAG AAT GGT GC-3′], its five base mismatch control [5′-GTA ATT ATT TAT AAT CGT CC-3′] and 3′ carboxytetramethylrhodamine (TAMRA) conjugated versions were synthesized as described (24). A 200 mg batch of SSO623 for *in vivo* studies was prepared by Avecia (Milford, MA, USA). A 2′-O-Me gapmer phosphorothioate anti-MDR1 ASO (5′-CCATCccgacctcgcGCTCC-3′) [2′-O-Me modifications in capitals] and its scrambled control were obtained from Integrated DNA Technologies (Coralville, IA, USA). An SSO (5′-TGGTTCTTACCCAGCCGCCG-3′) that causes redirection of Bcl-x pre-mRNA splicing from Bcl-xL to –xS has been previously described (54). Cholesterol-modified siRNA targeting Enhanced Green Fluorescent Protein (EGFP) (5′-gccacaacgucuauaucau-3′) and its mismatch control were obtained from Invitrogen/Life Technologies (Carlsbad, CA, USA). RNA isolation and reverse transcriptase-polymerase chain reaction analysis (RT-PCR) for Bcl-x and for EGFP were performed as previously described (53). An Alexa 488-tagged monoclonal antibody to P-glycoprotein (Pgp) was from BD-Pharmingen (San Jose, CA, USA). Lipofectamine 2000, LysoTracker Green^®^ lysosomotropic dye, Alexa 488 labeled dextran and baculovirus expression systems (Organelle LightsTM) were obtained from Invitrogen/Life Technologies (Carlsbad, CA, USA). HeLaEGFP654 is a human cell line containing an enhanced EGFP reporter interrupted by an abnormal intron. HeLaLuc705 and the human melanoma line A375Luc705 contain a similarly structured luciferase reporter (24,53). In each of these cell lines, correct splicing and reporter expression can be restored by delivery of SSO623 to the nucleus. NIH-3T3-MDR is a mouse fibroblast cell line stably transfected with a complementary DNA coding for the human Pgp multi-drug transporter and was obtained from M. Gottesmann (National Cancer Institute).

### Compound libraries

The University of North Carolina (UNC) compound libraries used in this study as well as our general approaches to high-throughput screening have been previously described (55–58). The Southern Research Library, the source of the three hits studied here, is a 13,392 compound collection made available through a collaboration between UNC and the Southern Research Institute.

### High-throughput screening

Our high-throughput screen (HTS) utilized HeLaLuc705 cells. Delivery of an appropriate SSO to the nucleus corrects splicing and induces luciferase expression thus providing an easily interpreted positive readout (24,26). Cells were trypsinized, rinsed and suspended in Opti-MEM at 300 000 cells/ml; SSO623 was added to a final concentration of 100 nM. No lipid or polymer transfection agents were used in these assays. Opti-MEM (15 µl) followed by cell suspension (20 µl) was added to wells of 384-well plates. Cells were allowed to attach and incubate with the SSO for 16 h. During this 16 h period a set of positive control wells received chloroquine to a final concentration of 300 µM while a set of negative control wells received diluent. After the initial incubation the remaining wells received library compounds to a final concentration of 25 µM. Incubation with library compounds was at 37°C for 5 h at which time the cells were harvested and analyzed for luciferase induction. Library compounds that produced an induction of 50% that of the positive controls were considered positive in this assay and were further analyzed. Dose-response curves were developed comparing SSO623 with a mis-matched oligonucleotide. Compounds that fully discriminated active SSO from its mismatched control were considered to be legitimate hits.

### SSO, antisense and siRNA assays

Luciferase induction dose-response curves with normalization on cell protein were performed in a 24-well format as previously described (24,26). Splicing modulation of the EGFP654 reporter and of endogenous Bcl-X were monitored by RT-PCR using appropriate primers. Evaluation of hit compound effects on antisense actions involved ‘knockdown’ of MDR1 pre-mRNA and its protein product in multi-drug resistant mouse 3T3 cells. Cell surface expression of P-glycoprotein was monitored using an Alexa-488 conjugated monoclonal antibody and flow cytometry as previously described (53,59). Evaluation of effects on siRNA utilized a HeLa cell line stably transfected with an EGFP expression cassette. Cholesterol-conjugated siRNAs were used to attain sufficient uptake. Cells were incubated with the siEGFP or control siRNA, rinsed and then briefly exposed to hit compound. After further incubation expression of EGFP was monitored by flow cytometry. In cases where treatment with Lipofectamine 2000 was used as a positive control, the manufacturer's protocol was followed.

### Cytotoxicity

An Alamar Blue assay was used to measure cytotoxicity (60). Cells were incubated with hit compounds under the same conditions as used for dose-response assays. After removal of the hit compound, cells were further incubated for 24–72 h in complete medium and then tested.

### Confocal studies

Quantitative live cell confocal microscopy (61) was performed to examine the subcellular distribution of fluorescent oligonucleotide or of certain markers for endomembrane compartments. HeLa cells were transfected with baculovirus expression vectors for Green Fluorescent Protein (GFP) chimeras of marker proteins for several endomembrane compartments. The day following transfection, cells were incubated for 4 h with 300 nM TAMRA conjugated SSO 623 in OptiMEM after which the cells were washed and incubated in Dulbecco's modified Eagle's medium (DMEM) medium with 1% FBS. In some cases hit compounds were added during or after the incubation. Cells were imaged on an Olympus FV1000 MPE laser scanning confocal microscope with environmental chamber to maintain 37°C, 40% humidity and 5% CO_2_. We used 488 nm (GFP) and 559 nm (TAMRA) as laser lines and images were collected with a 60× oil immersion lens. Quantitation of co-localization of oligonucleotide and marker proteins utilized the Coloc2 plug-in in Image J. In some cases GFP markers for organelles were examined in cells previously fixed in 4% formaldehyde in phosphate buffered saline (PBS).

### Flow cytometry

Measurement of Pgp expression and Lysotracker Green accumulation were performed by flow cytometery using an LSR II cell analyzer (Becton-Dickenson, San Jose, CA, USA) as previously described (53).

### *In vivo* effects and toxicity

All animal procedures were in accordance with guidelines of the UNC Laboratory Animal Medicine Department and with federal guidelines. The EGFP654 transgenic mouse has been described previously (62,63). A reporter gene comprised of the EGFP coding sequence is interrupted by an aberrantly spliced intron. Delivery of an appropriate SSO to the nucleus of tissue cells will correct splicing leading to the expression of normal EGFP mRNA and protein. EGFP654 mice were administered 25 mg/kg SSO623 or mis-matched oligonucleotide in PBS by intra-peritoneal injection on two consecutive days. One day later the mice received 7.5 mg/kg UNC10217938A intravenously in a diluent of 5% PEG400, or diluent only. After 24 h the mice were euthanized and cardiac blood and tissue samples collected. Tissues for fluorescence microscopy to visualize EGFP were fixed in cold 1.5% paraformaldehyde in PBS and then processed for cryosectioning (62). Tissues for RNA analysis were collected from mice euthanized at 4 h and were quick frozen on dry ice. All procedures involving live animals were conducted by the UNC Animal Studies Core facility. Blood samples were analyzed by the UNC Animal Clinical Chemistry Core facility.

## RESULTS

### High-throughput screening

**Figure 1.**
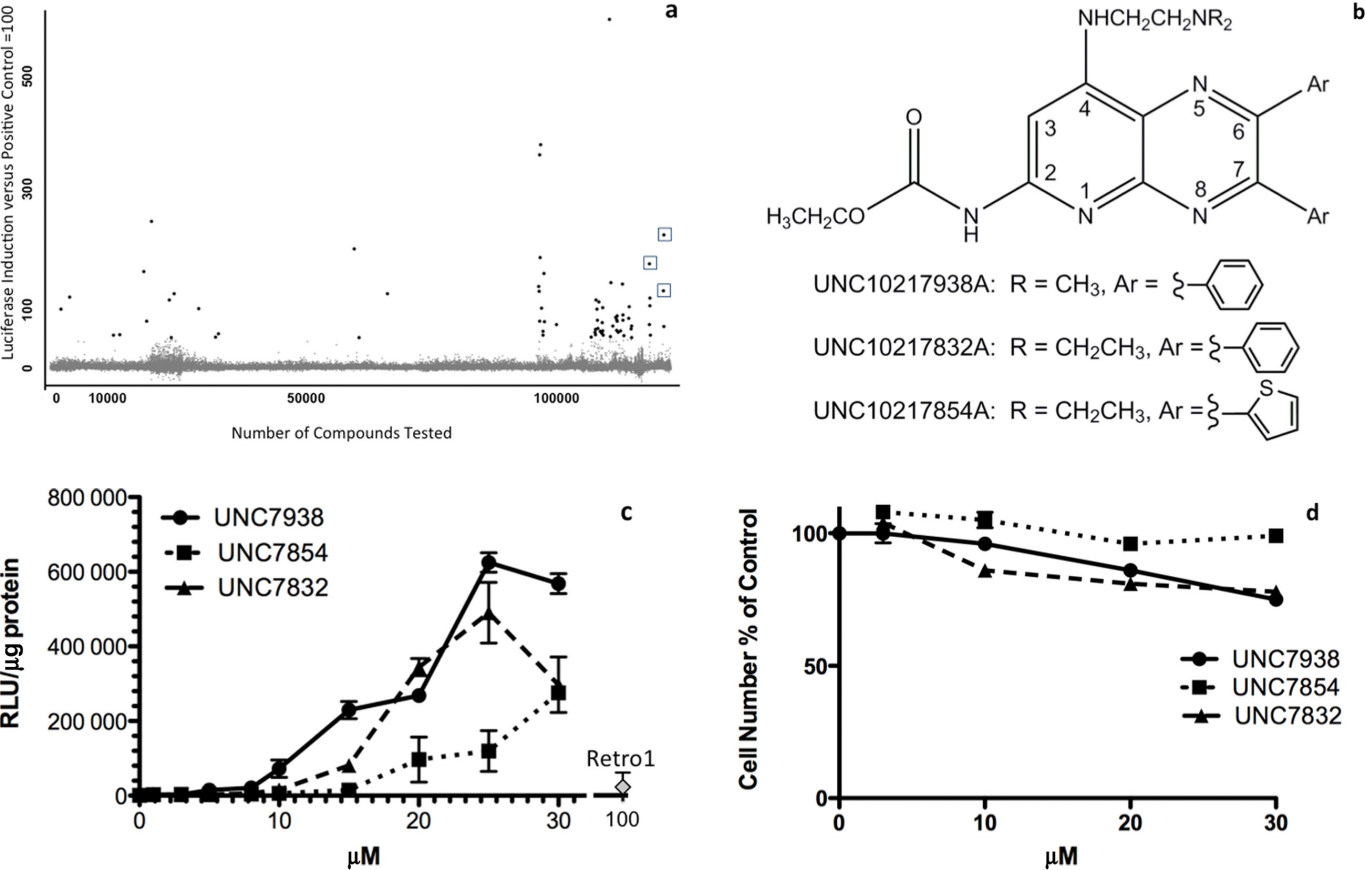
Screening and Chemistry of Hit Compounds. (**a**) *High-Throughput Screen Summary*. The abscissa indicates the number of compounds screened. The ordinate indicates the activity as percent of the positive control. Individual compounds are indicated as dots. Some of the dots represent false positives that did not discriminate SSOs from mismatched oligonucleotides. The three compounds chosen for investigation in this study are indicated by boxes. (**b**) *Structures of Hit Compounds*. (**c**) *SSO Dose-Response*. Selected hits were examined for their ability to enhance splice correction by SSO623 in the HeLa Luc 705 cell system. Cells were incubated in 24-well plates with 100 nM 623 or its mismatched control (MM) for 16 h in DMEM +10% FBS, rinsed and then treated with various concentrations of the hit compounds, or with 100 µM Retro-1, for 2 h. The cells were then rinsed and incubation continued for an additional 4 h in DMEM +10% FBS. Cells were rinsed twice in PBS and luciferase activity (RLU) and cell protein determined. Means +/− SE. *N* = 3. Mismatched controls were at baseline level on this scale and are not shown. (**d**) *Cytotoxicity*. Cells were exposed to hit compounds as in (c) then incubated for 24 h in DMEM plus 10% FBS and tested using the Alamar Blue cytotoxicity assay. Means +/− SE. *N* = 3.

Our high-throughput screening effort utilized a SSO (termed SSO623) and our previously described luciferase induction assay (24,26,53) optimized for screening. We screened >100,000 compounds from several libraries using a 384 well format. The *Z*-values were 0.8 or greater in all cases. The total number of hits was rather low, totaling only 67 or 0.04%. The majority of the hits were confirmed using the primary assay, but many also induced activity with a mismatched oligonucleotide and were designated as false positives since they made the splicesosome less discriminating and thus increased spontaneous splice correction; many of the strongest hits fell into this category. Following stringent secondary assays we identified three distinct series of compounds that met the following criteria: (i) they strongly increased luciferase induction by SSO623 but not a mismatched oligonucleotide; (ii) they were not toxic to cells at concentrations sufficient to substantially increase induction. We decided to initially pursue a series of 3-deazapteridine analogs because of their strong oligonucleotide enhancing effects. A depiction of the screen and the structures of three active compounds are shown in Figure 1a and b (active compounds, UNC10217938A, UNC10217832A, UNC10217854A, hereafter abbreviated as 7938, 7832, 7854). Several compounds with closely related structures were inactive in the screen suggesting that activity was due to specific molecular interactions rather than general physical properties (Supplementary Material Table S1, Figure S1).

### Dose-response, specificity and cytotoxicity relationships

The confirmed hits were re-tested for SSO-mediated luciferase induction and for cytotoxicity in a 24-well format that allows for normalization based on cell protein. As seen in Figure 1c the compounds strongly enhanced luciferase induction in HelaLuc705 cells when used in the 5–25 µM range and were substantially more effective and potent than Retro-1. For example, as compared to SSO alone, 7938 provided a 60-fold enhancement at 10 µM and 220-fold at 20 µM, in contrast to a 11-fold enhancement for 100 µM Retro-1. Mis-matched oligonucleotide had no effect in the presence of the hit compounds thus demonstrating specificity. As indicated in Figure 1d, over 24 h only modest cytotoxicity was manifested below 20 µM. However, continuous protracted exposure to compound resulted in increased toxicity, as might be expected (Supplementary Material Figure S2a). Pre-loading the cells with oligonucleotide did not alter the cytotoxicity of the hit compound (Supplementary Figure S2b). These compounds displayed a rapid onset of action (Supplementary Materials Figure S3), while the magnitude of the luciferase induction effect was only slightly less than that attained with use of a commercial cationic lipid transfection agent (Supplementary Figure S4). We also examined the ability of the compounds to enhance SSO-mediated splicing of an endogenous message (Bcl-x) and found them effective in this context (Supplementary Figure S5). When the amount of SSO was varied in the presence of a constant concentration of compound 7938, strong induction effects were observed with as little as 3 nM oligonucleotide (Supplementary Figure S6a). Thus, in summary, the hit compounds can strongly enhance the actions of SSOs at compound concentrations that display only modest cytotoxicity.

### Effects on antisense and siRNA oligonucleotides

An important issue is whether the hit compounds directly affect the splicing process versus affecting the delivery of SSOs to the nucleus where splicing takes place. To address this issue we examined compound effects on the actions of an ASO that acts on pre-mRNA via RNase H in the nucleus and on a siRNA that acts via the RISC complex in the cytosol. The ability to influence the actions of all three types of oligonucleotide would indicate that the hit compound affects delivery rather specific molecular events.

**Figure 2.**
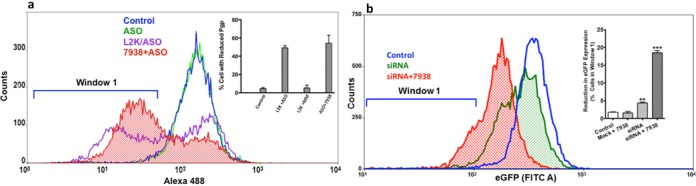
Effects on Antisense and siRNA. (**a**) *Antisense*. Pgp overexpressing NIH 3T3-MDR cells were treated with 100 nM anti-MDR1 ASO or a mismatched (MM) control for 16 h in DMEM +1% FBS. Cells were rinsed and then treated with 10 µM 7938 for 2 h. The compound was removed and the cells further incubated for 48 h. Expression of Pgp on the cell surface was determined using Alex488 labeled anti-Pgp with quantitation by flow cytometry. Treatment with ASO or MM complexed with Lipofectamine 2000 (L2K) were controls. All profiles were taken after initial gating on live cells. Ordinate, cell counts; Abscissa, Alexa 488 fluorescence. The profiles shown are typical of several independent experiments. In the inset the ordinate is the percentage of cells in Window 1 (reduced Pgp expression). Means +/− SE. *N* = 4. (**b**) *siRNA*. HeLa cells constitutively expressing EGFP were incubated with a cholesterol conjugated siEGFP or an irrelevant siRNA (mock) (both100 nM) in DMEM +1% FBS for 16 h and then treated with 10 µM 7938 for 2 h. After a further 24 h incubation EGFP expression was measured by flow cytometry. The main panel shows typical cytometry profiles. The inset shows the percent cells in window 1 (reduced EGFP expression). Mean +/− SE. *N* = 3. *** represents *P* < 0.001 compared to control.

To test the influence of hit compounds on antisense we examined the ability to enhance ‘knockdown’ of the P-glycoprotein multidrug transporter. As seen in Figure 2a, treatment of multi-drug resistant 3T3 cells with ASO alone had little effect, while use of a commercial cationic lipid transfection agent significantly enhanced the antisense action. Treatment of cells with 7938 also strongly enhanced ASO action to an extent comparable to that produced by the cationic lipid. In all cases only a portion of the cell population was affected as is typical in this model (59). When the amount of ASO was varied in the presence of a constant concentration of 7938, strong effects were observed with as little as 5 nM oligonucleotide (Supplementary Figure S6b). Compounds 7832 and 7854 also enhanced the effects of an anti-MDR1 ASO but had no effect on mismatched oligonucleotides (Supplementary Figure S7). Strong antisense effects were attained at concentrations of enhancing compounds that displayed little cytotoxicity (Supplementary Figure S8).

Since conventional siRNA is unstable in cell culture media and is poorly taken up by cells, we used a cholesterol-conjugated siRNA to test effects of the enhancing compound. As seen in Figure 2b, use of 7938 enhanced the ability of a siEGFP to reduce levels of EGFP in a cell line that stably expresses this reporter. A cholesterol-conjugated irrelevant control siRNA had no effect in the presence or absence of 7938.

These studies indicate that the hit compounds can enhance the pharmacological effects of several types of oligonucleotides that have distinct mechanisms of action. This supports the concept that these compounds act by influencing the intracellular trafficking and delivery of oligonucleotides rather than their direct actions.

### Effects on receptor-targeted oligonucleotide conjugates

**Figure 3.**
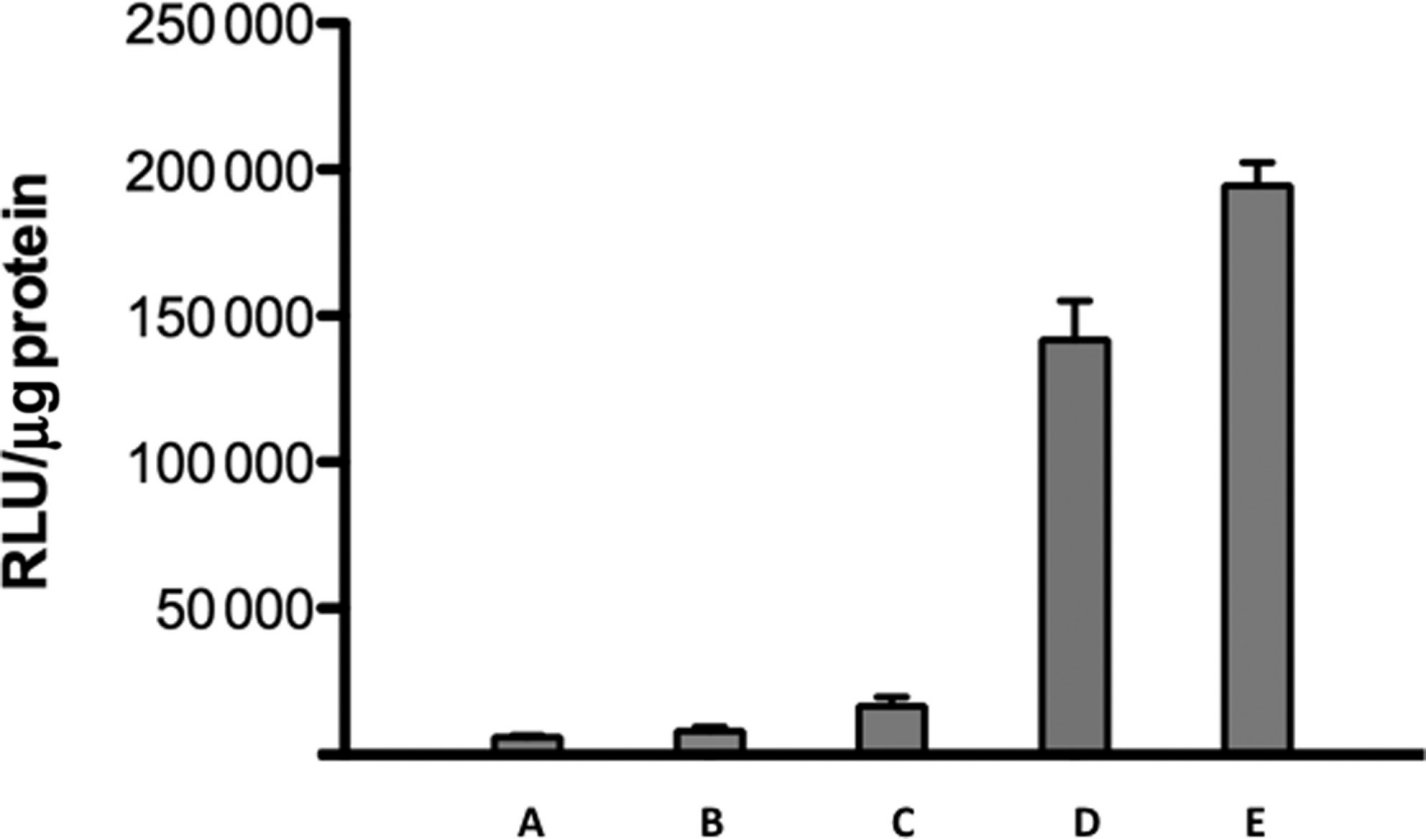
Effects on Receptor-Targeted Oligonucleotides. A375Luc705 cells were treated with 100 nM RGD-SSO-albumin conjugate for 16 h and subsequently treated with Retro-1 (100 µM) or 7938 (5, 10 µM) for 2 h. After removal of the compound the cells were incubated for a further 24 h and then assayed for luciferase and cell protein. (A) Control; (B) RGD-SSO conjugate only; (C) RGD-SSO conjugate plus Retro1; (D) RGD-SSO conjugate plus 5 µM 7938; (E) RGD-SSO conjugate plus 10 µM 7938. Means +/− SE. *N* = 3.

Our laboratory has pursued the use of ligand-oligonucleotide conjugates to attain receptor-selective targeted delivery (25,27,28). Thus we were interested in whether our compounds would affect oligonucleotides that enter cells by receptor-mediated endocytosis. We used a previously described (27) multivalent conjugate comprised of several RGD peptide-conjugated morpholino SSOs further conjugated via bioreversible links to serum albumin as a carrier. This conjugate displays efficient and selective uptake by cells that express RGD-binding integrins and modest but distinct splice correction effects (27). We examined luciferase induction in αvβ3-expressing A375 melanoma cells that contain the Luc705 expression cassette. As seen in Figure 3, treatment with 100 µM Retro-1 increased the ability of the conjugate to induce luciferase; however, 7938 at 5 or 10 µM had a far larger impact. Thus 7938 can substantially enhance effects of receptor targeted oligonucleotide conjugates. Additionally, this experiment illustrates that the compound can enhance effects of uncharged morpholino oligonucleotides as well as negatively charged oligonucleotides.

### Effects on the cellular endomembrane system and on subcellular distribution of oligonucleotide

The results mentioned above suggested that the hit compounds affect the delivery of oligonucleotide to the cytosol and nucleus. Subsequent to cell uptake, oligonucleotides traffic through multiple endomembrane compartments (12,39). Our previous work and that of others has shown that typical antisense and SSO oligonucleotides primarily accumulate in late endosomes and lysosomes (27,48,53). Thus we were interested in studying the impact of the hit compounds on those organelles and on the intracellular distribution of oligonucleotide. We used baculovirus expression vectors for GFP chimeras of well-known marker proteins for distinct endomembrane compartments to visualize those compartments. This included GFP-Rab7a for late endosomes, GFP-LAMP1 for lysosomes and GFP-N-acetylgalactosaminyltransferase 2 for Golgi apparatus (42,43). Transfection conditions were chosen so as to produce a mixture of untransfected cells and cells expressing the GFP chimeras in order to avoid overexpression artifacts.

**Figure 4.**
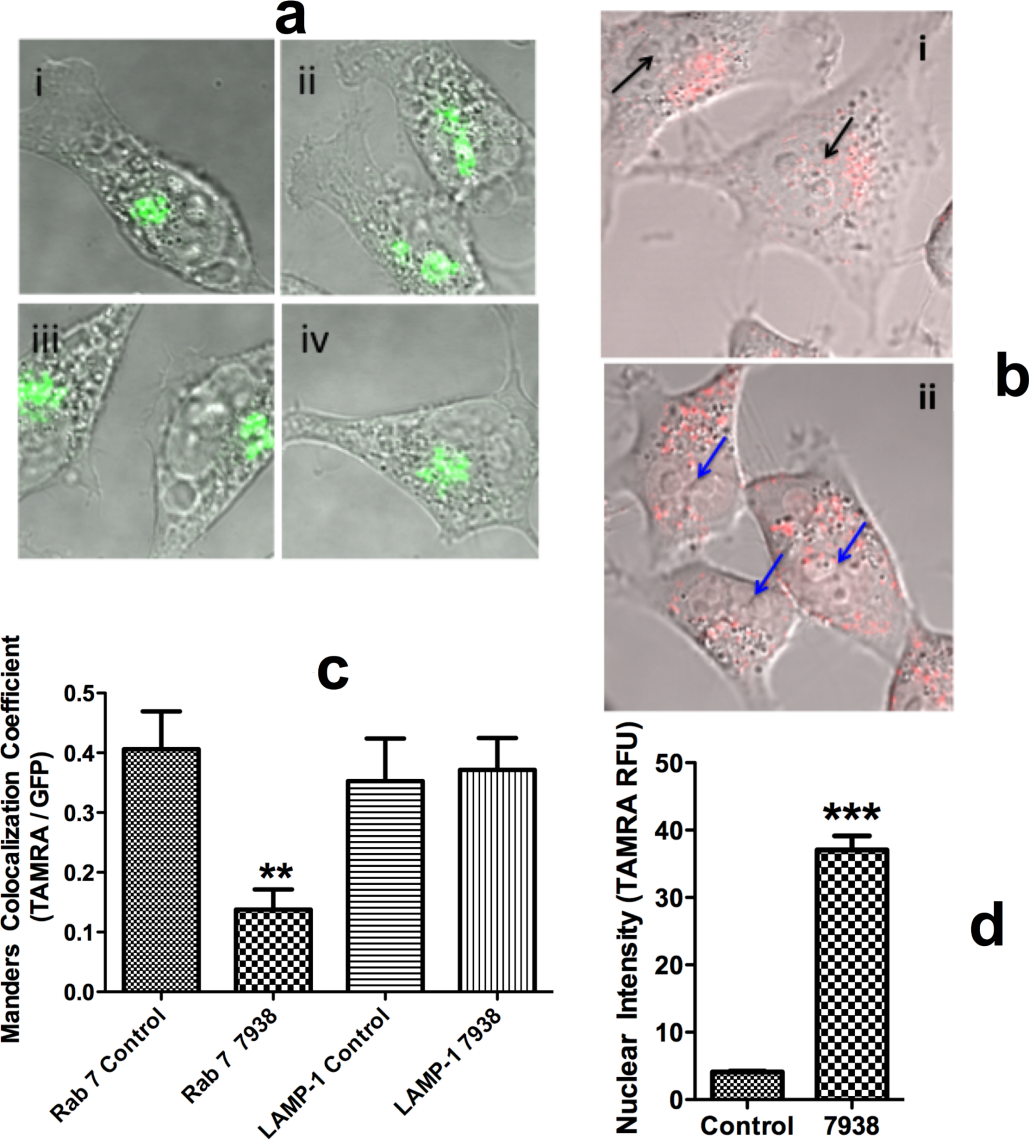
Effects on Cell Morphology and Subcellular Distribution of Oligonucleotide. (**a**) *Cell Morphology*. HeLa cells were transfected with an expression vector for GFP-N-acetylgalactosaminyltransferase 2 (Golgi marker). Cells were then treated with 10 µM 7938 for 30 min in DMEM +1% FBS at 37°C or maintained as controls. Cells were fixed in 4% formaldehyde in PBS for 10 min and then rinsed. Cell images were acquired by confocal microscopy. Untreated controls (i, ii) and treated with 7938 (iii, iv). (Panels **b**–**d**) HeLa cells were transfected with baculovirus expression vectors for GFP-Rab7a (late endosome marker) or GFP-LAMP-1 (lysosome marker). The cells were then incubated with 300nM SSO 623-TAMRA for 16 h. Cells were then treated with 10 µM 7938 for 1 h or maintained as controls. Live cells were imaged using a confocal microscope with environmental stage. (b) *Oligonucleotide Distribution*. Superimposed fluorescence/DIC images. The red dots indicate TAMRA-623 in vesicles. (i) Control cells; black arrows indicate ‘empty’ nuclei. (ii) Cells treated with 7938; blue arrows indicate nuclei with increased TAMRA fluorescence. (c) *Quantitation of Colocalization*. Co-localization of GFP and TAMRA oligonucleotide was quantitated using the Image J Coloc2 plug-in and expressed as the Manders correlation coefficient. Individual cells were analyzed and the data summated. Means and standard errors shown. *N* = 9–20. ** represents *P* < 0.002 compared to Rab 7 control. (d) *Nuclear Accumulation*. TAMRA fluorescence intensity in the nucleus was quantitated using Image J. Means and standard errors shown. *N* = 55–70. *** represents *P* < 0.001 compared to control.

As seen in Figure 4a, treatment of cells with 7938 had little effect on the compact organization of the Golgi apparatus. The late endosome and lysosome compartments are more diffusely distributed but we did not observe major changes in the appearance of those compartments, nor in the number of well-defined vesicles, upon treatment with 7938 (Supplementary Figure S9). Thus, at least at the descriptive level, 7938 did not cause substantial disruptions of the overall organization of the subcellular organelles examined. A SSO labeled at the 3′ position with a TAMRA fluorophore was used to visualize 7938 effects on the subcellular distribution of oligonucleotide. Live cells were observed using a confocal microscope with environmental stage before and after addition of 7938. As seen in Figure 4b, in cells that seem healthy and with normal morphology, treatment with 7938 led to a partial redistribution of oligonucleotide from endomembrane compartments to the nucleus. However, most of the oligonucleotide remained within endosomes. To further explore oligonucleotide distribution we quantitated the co-localization of GFP and TAMRA (61,64). As well, we quantitated the TAMRA fluorescence signal per unit area within the nucleus. As seen in Figure 4c, exposure of cells to 7938 led to a major reduction in co-localization of the TAMRA-oliogonucleotide with the late endosome marker Rab7, but had little effect on co-localization with the lysosomal marker LAMP-1. This suggests that 7938 primarily affects oligonucleotides in late endosomes. As seen in Figure 4d, reduction of co-localization of the TAMRA and Rab7 signals was accompanied by an increase in accumulation of TAMRA-oligonucleotide in the nucleus. Thus Figure4b–d suggest that 7938 causes partial release of oligonucleotide from late endosomes to the cytosol followed by nuclear accumulation. In other studies we used a lysosomotropic dye to further probe possible effects of 7938 on lysosomes. Lysotracker Green primarily accumulates in the low pH lysosomal compartment; thus permeabilization of the lysosome membrane would disrupt the pH gradient and inhibit Lysotracker uptake by cells. As seen in Supplementary Figure S10, concentrations of 7938 that are effective in enhancing oligonucleotide actions have almost no effect on Lysotracker Green accumulation suggesting that there are very limited effects on the lysosomal membrane. Another question is whether the enhancing compounds can also cause the release of other large molecules from endomembrane compartments. In an initial experiment we examined the effects of 7938 on the subcellular distribution of a fluorescent dextran having a molecular mass similar to that of an oligonucleotide. As seen in Supplementary Figure S11 the dextran was partially released from endosomes subsequent to treatment with 7938.

### *In vivo* studies

**Figure 5.**
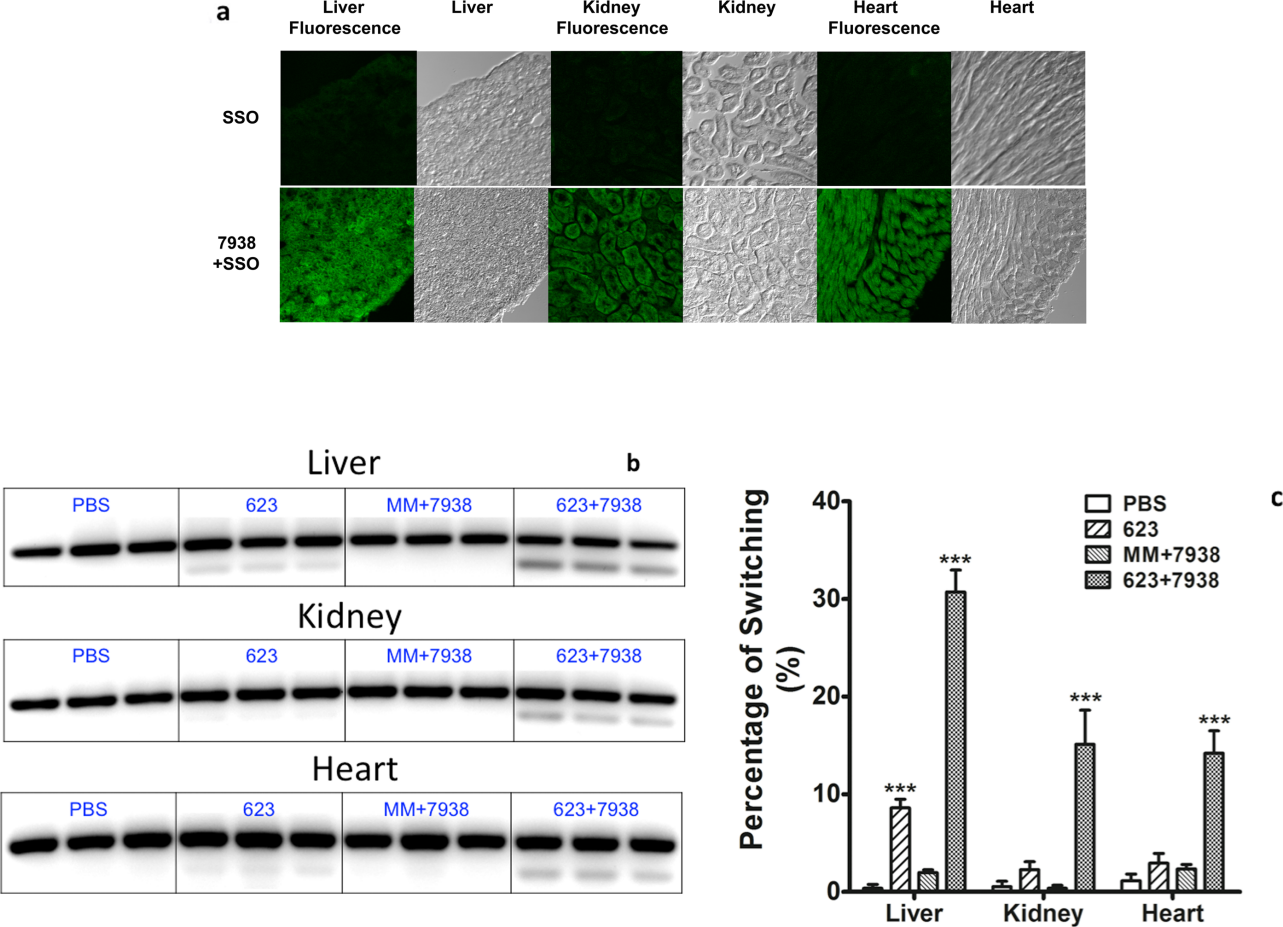
*In Vivo* Effects. (**a**) *EGFP Expression by Fluorescence Microscopy*. EGFP654 transgenic mice were treated with PBS, SSO623 or a mismatched control (MMSSO) followed by treatment with 7938 or diluent, as described in ‘Materials and Methods’ section. Cryosections of tissue samples were observed by fluorescence microscopy. Selected images from liver, kidney and heart are shown (additional images are shown in Supplementary Figure S12). The images shown were obtained 24 h after treatment with 7938. (**b**) *RT-PCR of EGFP mRNA*. EGFP654 mice were euthanized 4 h after injection of 7938. Tissues from three mice were processed for RT-PCR as described in ‘Materials and Methods’ section. The lower band is correctly spliced EGFP mRNA. The upper band is unspliced RNA. (**c**) *Quantitation of RT-PCR*. Gel bands in (b) were quantitated using a Typhoon imaging system. Percent of switching is the ratio of lower band to upper band × 100. *** difference from PBS control significant at the 0.001 level.

In order to determine whether the strong enhancing effects of our hit compounds that were seen in cell culture would also be observed *in vivo* we tested the effectiveness of compound 7938 using a transgenic mouse model that is responsive to SSOs (62,63). In the EGFP654 transgenic line a reporter gene comprised of the EGFP coding sequence is interrupted by an aberrantly spliced intron. Effective delivery of an appropriate SSO to the nucleus of tissue cells will correct splicing leading to expression of normal EGFP mRNA and protein in that tissue. We visualized EGFP expression by fluorescence microscopy of tissue cryosections and evaluated correction of EGFP pre-mRNA splicing by RT-PCR. For the *in vivo* study we extrapolated our cell culture data to estimate a dose of 7938 that might be effective and non-toxic. The effect of a single administration of 7938 is likely to be transient, while the half-life of EGFP protein is substantially longer than that of its mRNA (65,66), thus we monitored RNA at 4 h and protein at 24 h after 7938 administration. As seen in Figure 5a, systemic treatment with SSO623 followed by administration of 7938 produced distinct increases in EGFP fluorescence in liver, kidney and heart. Fluorescence was observed in the predominant cell type in each tissue including hepatocytes, kidney tubule cells and cardiac muscle cells. In contrast, systemic treatment with SSO623 alone produced very modest increases in fluorescence in these tissues. A broader representation of the EGFP induction is provided in Supplementary Figure S12. This shows that skeletal muscle was not strongly affected and also that the combination of mis-matched oligonucleotide and 7938 had no effect. As seen in Figure 5b and c, correctly spliced EGFP message was found in liver, kidney and heart, paralleling the observations on tissue sections. The dose of 7938 used in this experiment did not result in acute toxicity to the EGFP654 mice as indicated by the lack of significant changes in blood chemistry parameters (Supplementary Table S2). Additionally, a 7-day toxicity study done in C57BL/6 mice at doses that overlap those used in the experiment of Figure 5 showed no significant evidence of toxicity (Supplementary Table S3).

## DISCUSSION

Recent work has shown that most of the oligonucleotide taken up by cells accumulates in various endomembrane compartments especially late endosomes and lysosomes (40,48,53). The cellular endomembrane system is highly dynamic with numerous membrane fusion and disjunction events occurring as materials are trafficked throughout the cell (42,44). These events are regulated by a plethora of endomembrane-associated proteins. Thus, intracellular trafficking could potentially be influenced by small molecule drugs that affect endomembrane proteins; however, there has been relatively little work done on the chemical biology of the endomembrane system.

In this context, we performed a phenotypic HTS to identify small molecules that enhance oligonucleotide actions, with the expectation that some of these molecules might act by affecting oligonucleotide intracellular trafficking and/or release from endosomes. One set of compounds that emerged from this screen are the 3-deazapteridine analogs typified by UNC10217938A. When used in the low micromolar range in cell culture, these compounds are very effective in enhancing the actions of oligonucleotides while displaying modest cytotoxicity. Since they affect antisense, splice switching and siRNA oligonucleotides, it is clear that these compounds influence intracellular delivery of the oligonucleotide rather than the basic molecular mechanism involved in each case.

Our studies suggest that 7938 selectively releases oligonucleotide from late endosomes thus allowing increased access to the cytosol and nucleus. However, only a small fraction of the total endosomal oligonucleotide is released. At this point it is not clear whether a specific sub-set of endosomes is affected or if all late endosomes are affected to a limited degree. The steep dose-response curves suggest a threshold effect for oligonucleotide release rather than a more gradual process. The selective action of 7938 on late endosomes differentiates its mechanism from that of lysosomotropic compounds and from polymers that act by the ‘proton sponge’ effect (67). In contradistinction to those agents, which physically disrupt lysosomes and late endosomes due to pH changes and swelling, we hypothesize that 7938 and its close analogs act on a specific target in the endomembrane system. While the presumption is that the target is a protein, it remains possible that membrane lipids may be involved since endomembrane compartments are known to contain unique lipid constituents (68) that could be the basis for the observed compartmental selectivity. There are several ways in which our hit compounds could cause release of oligonucleotide to the cytosol. One would be an overall destabilization of late endosome membranes. However, there are other possible mechanisms including an increase in the rate or extent of tubulation and budding of carrier vesicles, or defects in the process of vesicle fusion (39). Interestingly other large molecules such as dextrans can also be released from endomembrane compartments by these compounds.

Our initial *in vivo* experiments demonstrated that 7938 can enhance the actions of a SSO in a transgenic mouse model without significant acute toxic effects. Only partial correction of splicing was attained, however, we observed correction of splicing in heart and kidney, tissues that are usually refractory to the type of SSO used in our studies (63). Thus, compounds such as 7938 may extend the range of tissues that can be affected by oligonucleotides.

At high concentrations the hit compounds are cytotoxic. This is not unexpected since disruption of trafficking and/or release of endomembrane contents could be deleterious. In particular disruption of lysosomes is known to cause activation of the inflammasome and generation of inflammatory mediators (69). Thus the observation that the hit compounds primarily affect late endosomes rather than lysosomes may be advantageous. Analogs of 3-deazapteridine can affect dihydrofolate reductase and tubulin (70) and these activities may also contribute to toxicity.

Our investigations demonstrate that it is possible to use high-throughput screening to find small molecules that strongly enhance the pharmacological effects of oligonucleotides, although such molecules are rare. The 3-deazapteridine hits that initially emerged from our screen display a rather narrow gap between effective and toxic concentrations and are effective only in the micromolar range. It would be desirable to have compounds that are effective in the nanomolar range both for potential therapeutic development and for pursuing the identity of molecular targets. Thus through additional screening and synthetic efforts we are currently seeking compounds with greater potency coupled with reduced toxicity. Such compounds may eventually make an important contribution to oligonucleotide-based therapeutics by providing an additional approach to oligonucleotide delivery, one that complements existing approaches such as use of nanocarriers.

## SUPPLEMENTARY DATA

Supplementary Data are available at NAR Online.

SUPPLEMENTARY DATA
