# Supplementary material for: High-throughput screening identifies small molecules that enhance the pharmacological effects of oligonucleotides
Source: Nucleic Acids Res. 2015 Feb 6;43(4):1987–96. doi: 10.1093/nar/gkv060 (PMC4344505; doi:10.1093/nar/gkv060)
Supplement: SUPPLEMENTARY DATA [file supp_43_4_1987__index.html]

High-throughput screening identifies small molecules that enhance the pharmacological effects of oligonucleotides — High-throughput screening identifies small molecules that enhance the pharmacological effects of oligonucleotides — SUPPLEMENTARY DATA 

# High-throughput screening identifies small molecules that enhance the pharmacological effects of oligonucleotides

## SUPPLEMENTARY DATA

**Files in this Data Supplement:**

- SUPPLEMENTARY DATA
