# Supplementary material for: High-throughput screening identifies small molecules that enhance the pharmacological effects of oligonucleotides
Source: Nucleic Acids Res. 2015 Feb 6;43(4):1987–96. doi: 10.1093/nar/gkv060 (PMC4344505; doi:10.1093/nar/gkv060)

**Supplementary Figures and Tables**

Supplementary Table 1. Inactive Analogs. Several closely related analogs of the active compounds were essentially inactive in the initial luciferase induction screen (<5% of the effect of UNC10217938A). These compounds were not toxic to cells at the concentration tested since basal levels of luciferase activity were maintained. The compounds are shown with both their original Southern Research Institute designations and the short form UNC designation. Additional data on compounds 7704 and 8428 is provided in Supplementary Figure S1. Compound 8722 is no longer available for testing.


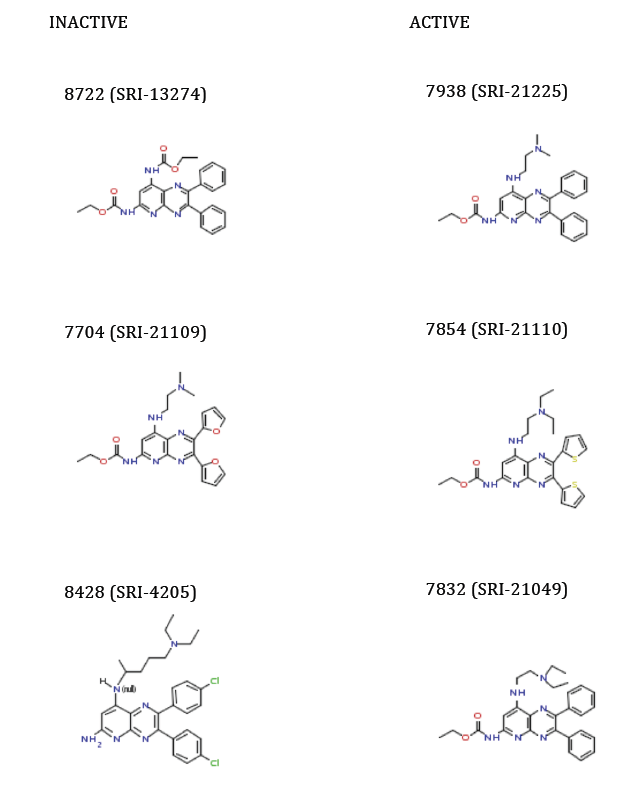


Supplementary Table 2. In Vivo Toxicity Tests. Cardiac blood samples from the mice used for the experiment in **Figure 5a** were analyzed for indications of kidney, liver or hematopoietic toxicity. Means +/- standard errors. N=5.

| **SAMPLE** | **BUN** | **ALT** | **AST** | **Hematocrit** | **Hb** | **Platelets** |
| --- | --- | --- | --- | --- | --- | --- |
| CONTROL | 22.5 +/-1.7 | 34.6+/-5.6 | 23.2+/-2.3 | 40.5+/-5.4 | 11.5+/-1.4 | 1068+/-113 |
| SSO | 23.2+/-0.8 | 37.2+/-6.0 | 24.8+/-2.4 | 40.5+/-1.6 | 11.5+/-0.6 | 922+/-335 |
| MMSSO +7938 | 17.8+/-3.3 | 36.2+/-3.7 | 24.8+/-10.6 | 38.8+/-3.5 | 11.2+/-0.9 | 1271+/-326 |
| SSO +7938 | 19.0+/-3.5 | 42.3+/-2.4 | 18.5+/-2.6 | 40.9+/-4.5 | 11.6+/-1.0 | 1345+/-311 |

BUN mg/dl, ALT U/L, AST U/L, Hematocrit %, Hemoglobin g/dl, Platelet x10^3^ /ul

BUN=blood urea nitrogen; ALT=alanine aminotransferase; AST=aspartate aminotransferase; Hb=hemoglobin

7938=7.5 mg/kg.

Supplementary Table 3. In Vivo Toxicity (7 day). Mice (C57BL/6) were injected IV with compound 7938 or with diluent only. Mice were observed and weighed daily. After 7 days mice were euthanized and cardiac blood samples analyzed for indications of kidney, liver or hematopoietic toxicity. Means +/- standard errors. N=4.

| **SAMPLE** | **BUN** | **ALT** | **AST** | **Hematocrit** | **Hb** | **Platelets** | **Δ Weight** |
| --- | --- | --- | --- | --- | --- | --- | --- |
| 7938 9 mg/kg | 24.3 +/- 4.0 | 20.5 +/- 3.0 | 32.5 +/- 37.0 | 42.3 +/- 2.0 | 14.3 +/- 0.7 | 760 +/- 350 | 0.8 +/- 0.5 |
| 7938 4.5 mg/kg | 25.0 +/- 2.2 | 28.3 +/- 4.0 | 23.5 +/- 7.0 | 41.4 +/- 1.2 | 14.0 +/- 0.4 | 791 +/- 330 | 0.6 +/- 0.3 |
| Control (diluent only) | 23.5 +/- 4.5 | 25.0 +/- 3.0 | 28.0+/- 10.0 | 46.0 +/- 0.8 | 14.2 +/- 0.2 | 864 +/- 98 | 0.9 +/- 0.7 |

BUN mg/dl, ALT U/L, AST U/L, Hematocrit %, Hemoglobin g/dl, Platelet x10^3^ /ul. Means +/- SE. N=4.

BUN=blood urea nitrogen; ALT=alanine aminotransferase; AST=aspartate aminotransferase; Hb=hemoglobin; Δ Weight= change in weight (g) from start of experiment.

Supplementary Figure S1. Test of Inactive Analogs. Two of the inactive or poorly active analogs shown in Supplementary Table 1 were tested versus compound 7938 in a luciferase induction assay using HeLa Luc705 cells and SSO623. The procedure was the same as that used in Figure 1c of the main text. Means and standard errors shown. N=3.


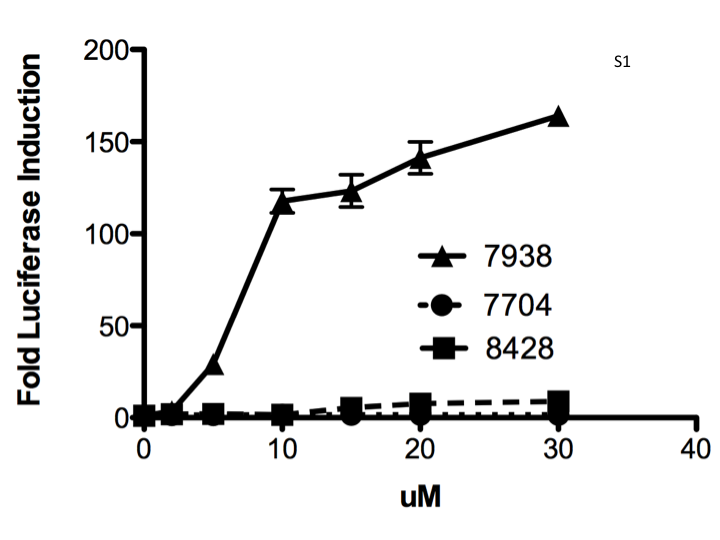


Supplementary Figure S2. Additional Cytotoxicity Experiments.

(a) *72 Hour Toxicity*. In one experiment HeLa Luc 705 cells were incubated with the indicated concentrations of compound 7938 for 2h in medium+ 10% serum followed by removal of the compound and further incubation in medium +10% serum for 72h. In a second experiment the cells were exposed to compound for the full 72h. Cytotoxicity was evaluated using the Alamar Blue assay. Means and standard errors shown. N=3.


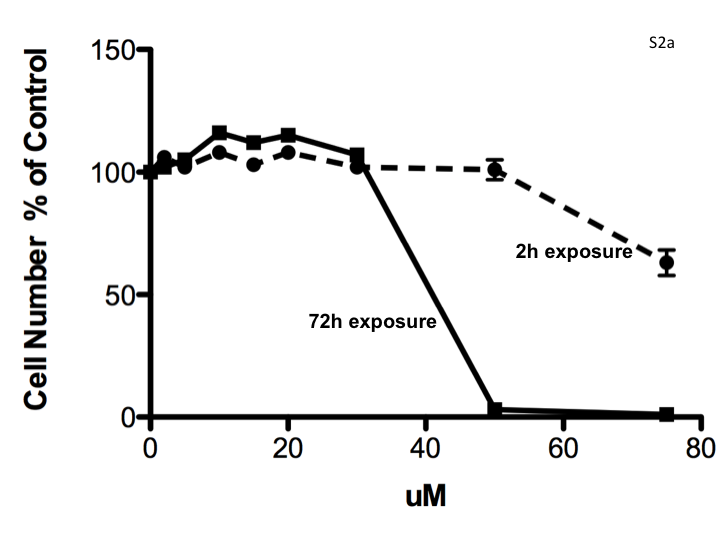


(b) *Effect of Pre-loading*. HeLa Luc 705 cells were preloaded with 100 nM SSO623 overnight and were then treated with various concentrations of 7938 for 2h in medium +10% serum. After 24 h further incubation cytotoxicity was evaluated using the Alamar blue assay. Means and standard errors shown. N=3.


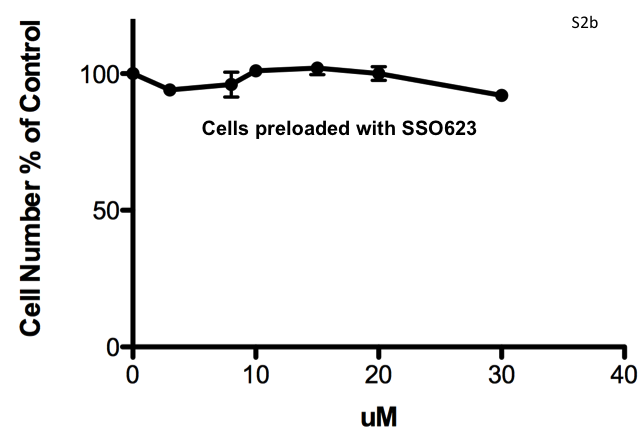


Supplementary Figure S3. Time-response. HeLa Luc705 cells were incubated with 100 nM SSO623 for 16 h, rinsed, and then exposed to UNC10217938A (10 uM) for various intervals. The compound was then rinsed away and incubation continued for a total of 6 h. Cells were then rinsed in PBS and luciferase activity and cell protein determined. Mean +/- SE. N=3.


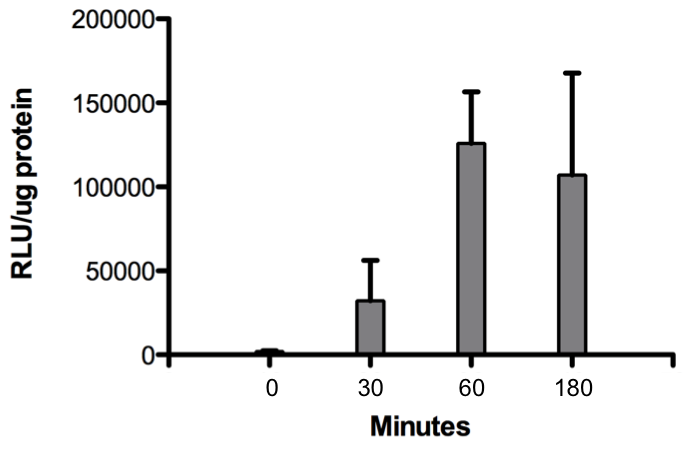
S3

Supplementary Figure S4. Comparison to Conventional Transfection. HeLaLuc705 cells were either transfected using 100 nM SSO623 or MM623 (mismatch) and Lipofectamine 2000 (L2K) (as per manufacturer’s protocol), or were incubated for 16 h with 100 nM SSO623 or MM623 followed by 2 h treatment with 10 um 7938. Mean +/-SE. N=3.

A 623 only; B MM623 only; C 623+L2K; D MM623 +L2K; E 623+7938; F MM623+7938.


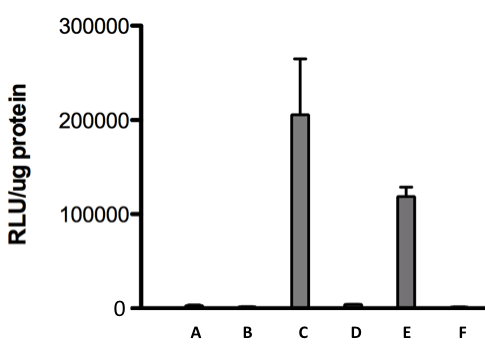
S4

Supplementary Figure S5. Alteration of Splicing of an Endogenous Gene. HeLa cells were incubated for 16 h with a SSO designed to cause splicing of the Bcl-x pre-mRNA to its short form (Bcl-xS) or with an irrelevant control (both 100 nM). After removal of the SSO some samples were treated for 2h with 10 uM of UNC10217938A, UNC10217832A, or UNC10217854A. After a further incubation cells were processed for RT-PCR and gel analysis. The blue arrows indicated the Bcl-xS band while the upper band is Bcl-xL.

1. HeLa cells (non-treated); 2. Bcl-SSO; 3. Irrelevant oligo + 10uM 7938; 4. Bcl-x SSO + 10uM 7938; 5. Irrelevant oligo+ 10uM 7832; 6. Bcl-x SSO + 10uM 7832; 7. Irrelevant oligo + 10uM 7854; 8. Bcl-x SSO + 10uM 7854.

S5


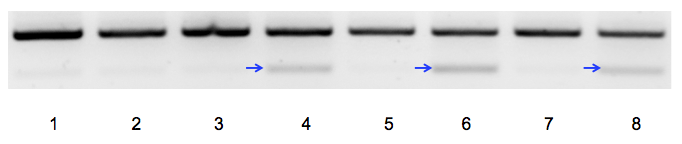


Supplementary Figure S6. Effect of Altering the Oligonucleotide Concentration.

(a). *SSO effects*. HeLa Luc705 cells were preincubated for 16 h with various concentrations of SSO623 or a mismatched control. After removal of the oligonucleotide the cells were treated with 10 uM 7938 for 2h and the compound then removed. After 4h further incubation the cells were harvested and analyzed for luciferase induction and cell protein. Means +/- SE. N=3.


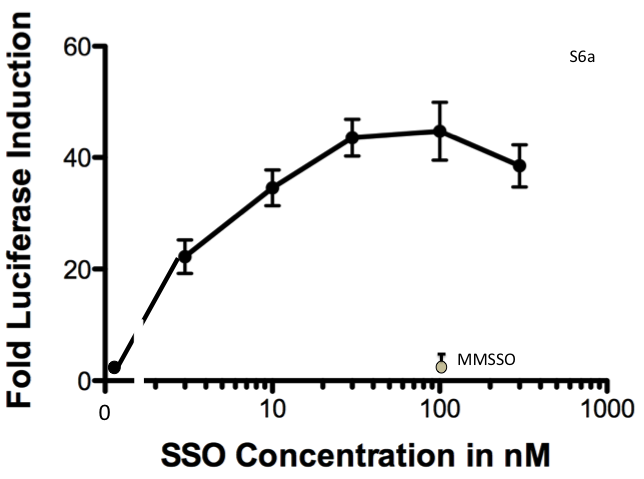


(b) *ASO Effects*. NIH-3T3-MDR cells were incubated for 16 with various concentrations of anti-MDR1 ASO. After removal of the oligonucleotide the cells were treated with 10 uM 7938 for 2h and the compound then removed. After 48h further incubation the cells were harvested and analyzed for expression of P-glycoprotein by flow cytometry. The data indicates the % of cells with Pgp levels in a window set to include 5% of the control untreated population (low expression). Means +/- SD. N=3.


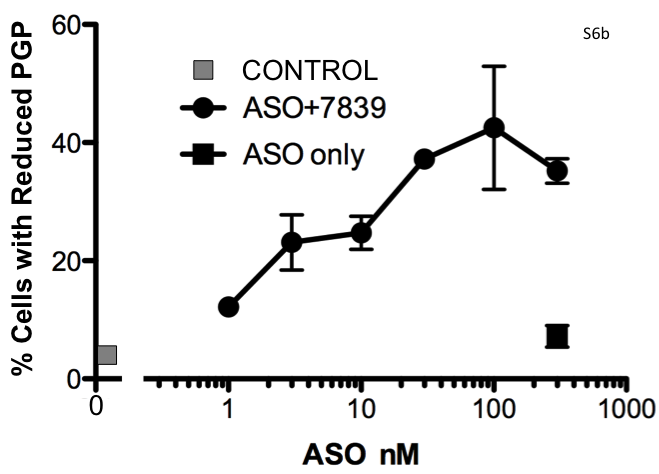


Supplementary Figure S7. Effects of Hit Compounds on ASO-mediated Reduction of Pgp Expression. NIH-3T3-MDR cells were incubated for 16 with 100 nM anti-MDR1 ASO or mismatched (MM) control. After removal of the oligonucleotide the cells were treated for 2h with 10 uM 7938 or 7832, or with 20 uM 7854, and the compounds then removed. After 48h further incubation the cells were harvested and analyzed for expression of P-glycoprotein by flow cytometry. The data indicates the % of cells with Pgp levels in a window set to include 5% of the control untreated population (low expression). Means of duplicate determinations shown.


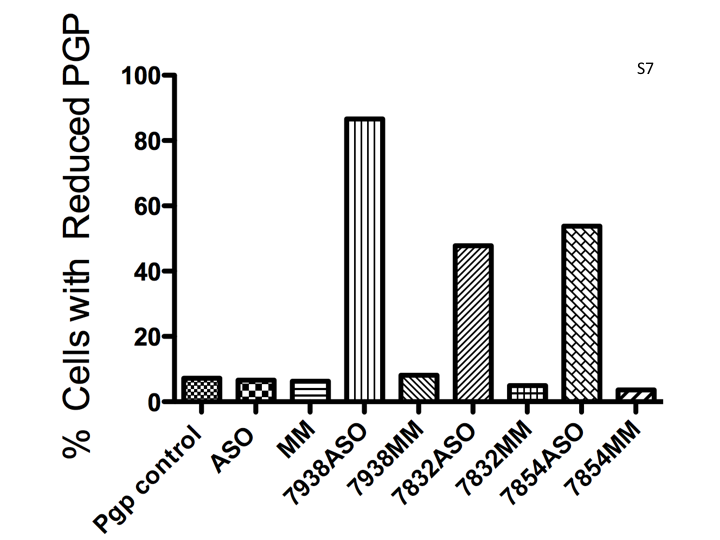


Supplementary Figure S8. Cytotoxicity of Oligonucleotide Enhancing Compounds in NIH-3T3-MDR cells.

Cells were incubated with various concentrations of 7938,7832 or 7854 for 2h in DMEM +1% FBS. Cells were rinsed and incubated a further 48h. Cytotoxicity was measured using an Alamar Blue assay. Mean+/- SE. N=3.


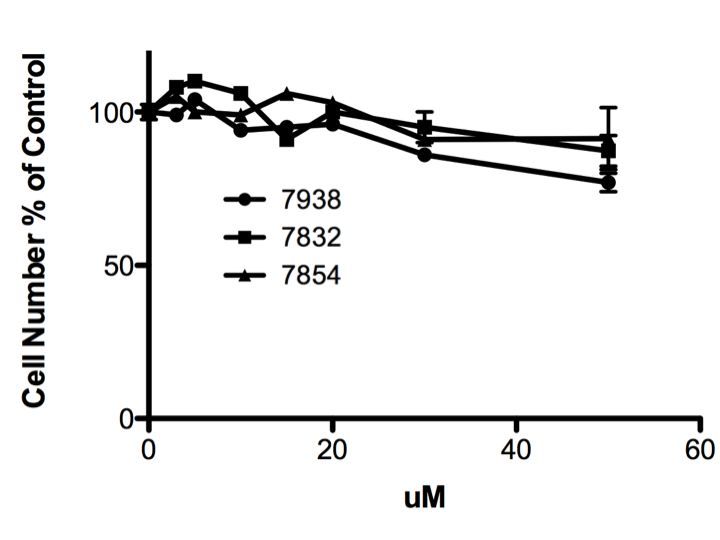


S8

Supplementary Figure S9. Lack of Effect of 7938 on the Morphology of Late Endosomes or Lysosomes.

(a) *Images.* HeLa cells were transfected with expression vectors for GFP-Rab7 (late endosome marker) or LAMP-1 (lysosome marker). Cells were treated with 10 uM 7938 or maintained as controls. After 30 min. incubation cells were fixed in 4% paraformaldehyde, rinsed, and then observed by confocal fluorescence microscopy. Late endosome control (i,ii), with 7938 (iii, iv). Lysosome control (v), with 7938 (vi).


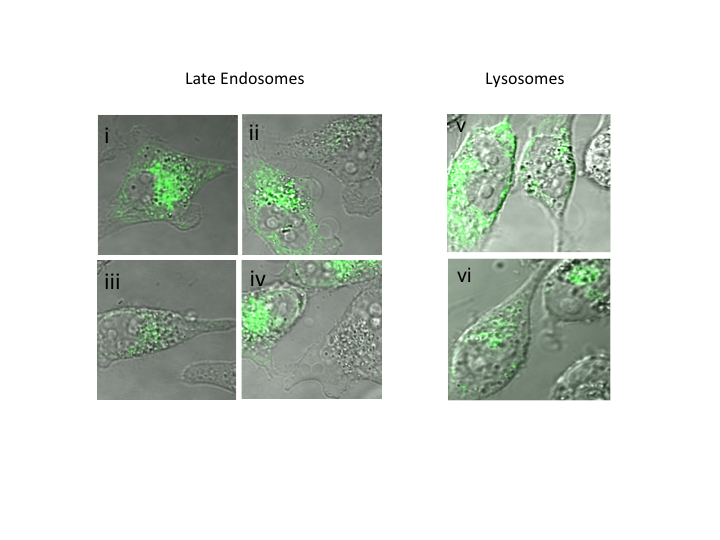


S9a

(b) *Number of Vesicles*. The number of late endosome (LE) or lysosome (LY) vesicles were counted visually in several cells with or without treatment with 10 uM 7938. Means+/- SE. N=5-6 cells.


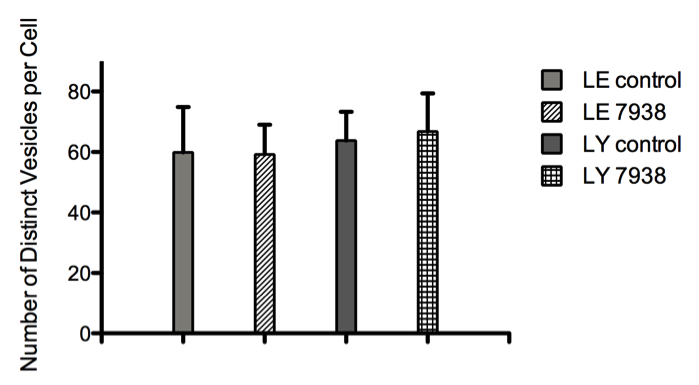


S9b

Supplementary Figure S10. Lack of Effect on Lysotracker Acccumulation. HeLa Luc705 cells were treated for 1h with 7938 at 5 or 10 uM, or with the V-ATPase inhibitor bafilomycin at 100 nM as a positive control. Cells were then exposed to 100 nM Lysotracker Green for 30 min and then rinsed and analyzed for Lysotracker accumulation by flow cytometry.

S10a. *Typical flow cytometry profiles*. Ordinate= number of cells; abscissa=color intensity of Lysotracker Green.


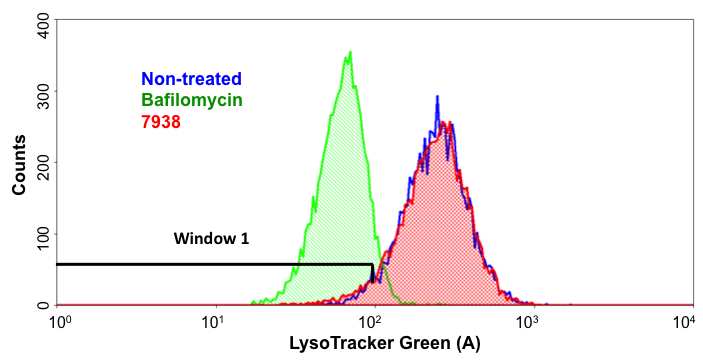
 S10b. *Quantitation*. Ordinate=% cells in Window 1 of S10a (reduced Lysotracker Green uptake). Chart shows means of duplicates; individual values as black circles. Control is cells treated with Lysotracker Green only.


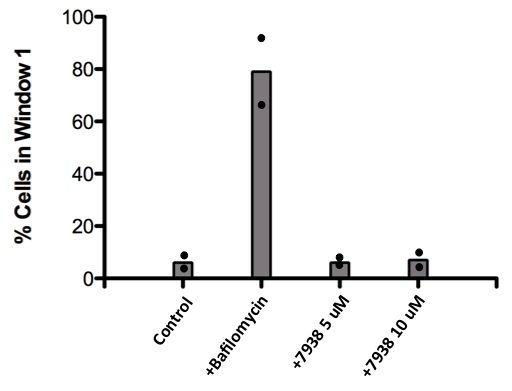


Supplementary Figure S11. Effect of 7938 on Release of Dextran From Endosomes.

HeLa cells (50,000) were seeded into glass-bottom dishes in medium +10% serum and incubated overnight at 37°C. Alexa 488-Dextran (10kD, 200µg/ml) was added and the cells were treated for 24 hours. Cells were washed with PBS and then treated with 10 μM 7938 for 2 h or maintained as controls. Live cells were imaged immediately after treatment using an Olympus FV1200 Confocal Microscope. Scale bar, 30µm.


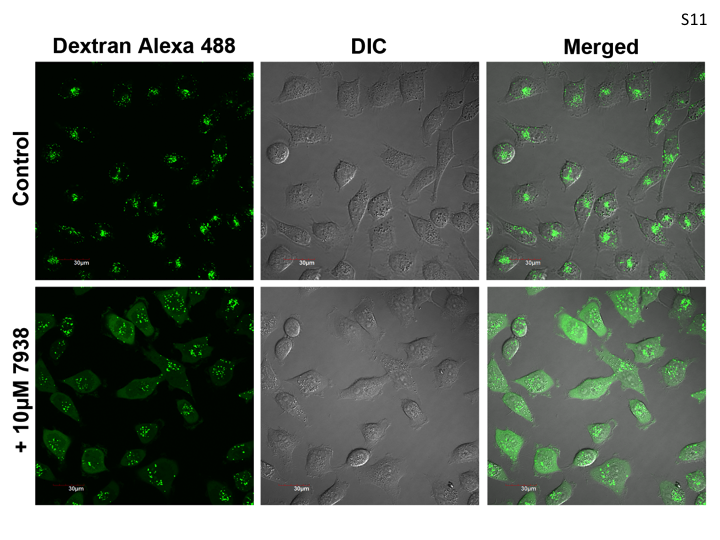


Supplementary Figure 12. EGFP Expression by Fluorescence Micros*copy*. EGFP654 transgenic mice were treated with PBS, SSO623 or a mismatched control (MMSSO) followed by treatment with 7938 or diluent, as described in Materials and Methods. Cryosections of tissue samples were observed by fluorescence microscopy. The images shown are representative of five mice per group and are samples that were obtained 24 h after treatment with 7938. Note that the samples marked MMSSO received 7938 as well as the oligonucleotide.


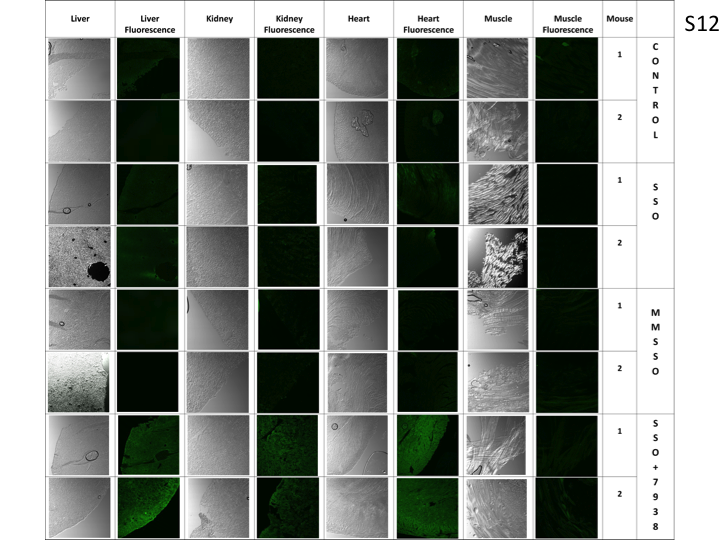

Supplement: SUPPLEMENTARY DATA [file supp_gkv060_nar-03269-y-2014-File013.docx]
